# A case report of the differential diagnosis of *Cellulosimicrobium cellulans*-infected endocarditis combined with intracranial infection by conventional blood culture and second-generation sequencing

**DOI:** 10.1186/s12879-020-05559-6

**Published:** 2020-11-26

**Authors:** Huifang Zhang, Chunyan He, Rui Tian, Ruilan Wang

**Affiliations:** 1 Emergency & Critical Care Department, Shanghai General Hospital, Shanghai Jiao Tong University School of Medicine, 650 New Songjiang Road, Songjiang District, Shanghai, China; 2 Laboratory Medicine Department, Shanghai General Hospital, Shanghai Jiao Tong University School of Medicine, Shanghai, China

**Keywords:** *Cellulosimicrobium cellulans*, Second-generation sequencing, Infectious endocarditis, Case report

## Abstract

**Background:**

*Cellulosimicrobium cellulans* is a gram-positive filamentous bacterium found primarily in soil and sewage that rarely causes human infection, especially in previously healthy adults, but when it does, it often indicates a poor prognosis.

**Case presentation:**

We report a case of endocarditis and intracranial infection caused by *C. cellulans* in a 52-year-old woman with normal immune function and no implants in vivo. The patient started with a febrile headache that progressed to impaired consciousness after 20 days, and she finally died after treatment with vancomycin combined with rifampicin. *C. cellulans* was isolated from her blood cultures for 3 consecutive days after her admission; however, there was only evidence of *C. cellulans* sequences for two samples in the second-generation sequencing data generated from her peripheral blood, which were ignored by the technicians. No *C. cellulans* bands were detected in her cerebrospinal fluid by second-generation sequencing.

**Conclusions:**

Second-generation sequencing seems to have limitations for certain specific strains of bacteria.

## Background

*Cellulosimicrobium cellulans*, formerly known as *Oerskovia xanthineolytica,* is a gram-positive filamentous bacterium found primarily in soil and sewage that rarely causes human infection, with only approximately 30 cases reported to date. In the reported cases, infections occurred mainly in immunocompromised hosts, patients with medically relevant implants, and newborns. We report a case of endocarditis and intracranial infection caused by *C. cellulans* in a 52-year-old woman with normal immune function and no implants in vivo. We found differences between the results of conventional blood culture and second-generation sequencing. Second-generation sequencing seems to have limitations for specific strains of bacteria.

## Case presentation

A 52-year-old rural housewife was admitted to our hospital with a sudden consciousness disorder and weakness of the right limb. One week prior, she was admitted to the local hospital with fever and headache and received intermittent infusion for 20 days (the specific treatment is not known), but her headache did not improve. The cranial magnetic resonance imaging and angiography (MRI + MRA) suggested an acute interstitial infarction in the left parietal lobe and a mildly abnormal electroencephalogram (EEG). The patient was previously healthy and had no history of diabetes, autoimmune disease, or hormone use.

**Fig. 1:**
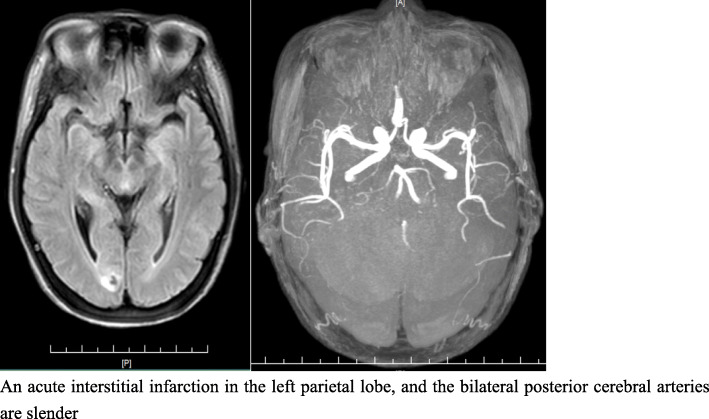
MRI + MRA images: An acute interstitial infarction in the left parietal lobe, and the bilateral posterior cerebral arteries are slender

Her body temperature on admission was 39.6 °C, with a Glasgow coma scale (GCS) score of 6 and bilateral pupil diameter of 5 mm with negative light reflection. Her neck was slightly stiff, a wind-like murmur during mitral valve systole was heard, and her skin and mucosa over her entire body were not visibly damaged. Laboratory examination showed peripheral blood leukocytes at 5.9 × 10^9^/L, neutrophils 83.7%, and C-reactive protein (CRP) at 29.7 mg/L. Cranial MRI showed abnormal signals in the right occipital parietal flap, which was considered to be a small focus of bleeding. Her bilateral posterior cerebral arteries were slightly slender (Fig. 1). The patient was diagnosed with infective endocarditis and an intracranial infection. Ceftriaxone sodium 2 g bid was given as empirical anti-infective therapy.

**Fig. 2:**
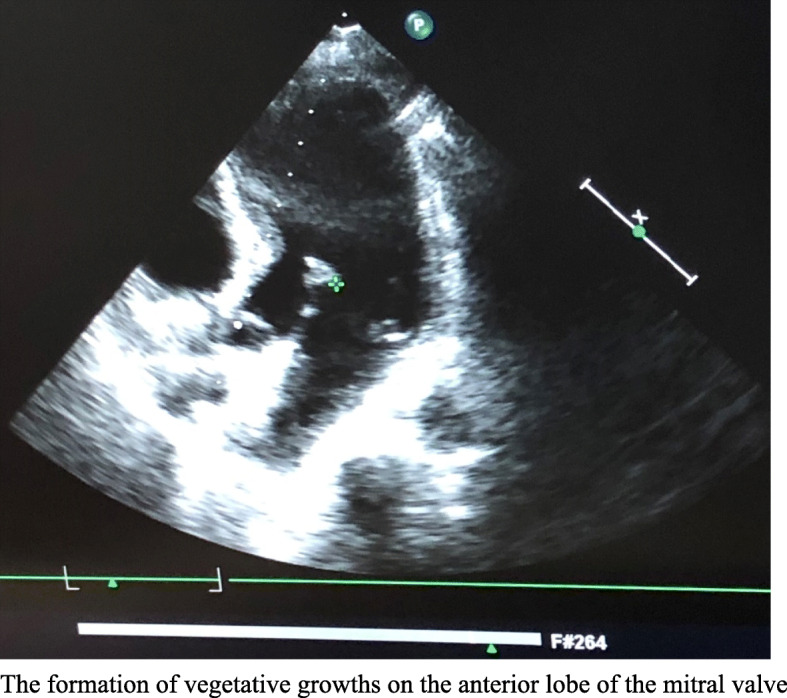
Transoesophageal echocardiography: The formation of vegetative growths on the anterior lobe of the mitral valve

After admission, lumbar puncture showed a cerebrospinal fluid (CSF) pressure of 150 mmH_2_O, cerebrospinal fluid leukocyte count of 622 × 10^6^/L, 90% multiple nucleated cells and 10% single nucleated cells, an erythrocyte count of 0 × 10^6^/L, protein quantification of 0.67 g/L, sugar of 2.9 mmol/L (peripheral blood sugar 6 mmol/L), lactate dehydrogenase (LDH) of 45.6 U/L, and chlorine of 127 mmol/L. Her cerebrospinal fluid smear was negative for bacteria, fungi, cryptococcus, or antibodies. Transoesophageal echocardiography showed the formation of vegetative growths on the anterior lobe of the mitral valve (Fig. 2). Vancomycin (1 g, q12h) combined with ceftriaxone sodium (2 g, q12h) and rifampicin (450 mg, bid) were given as empirical anti-infective treatment. No bacterial growth was seen in the cerebrospinal fluid culture after 24 h. Second-generation sequencing of her peripheral blood was negative for bacteria, fungi, viruses, parasites, and mycobacteria. The patient’s CD3+ T lymphocytes accounted for 77.2% of the total lymphocytes, with CD4+ T lymphocytes accounting for 49.72%, and CD8+ T lymphocytes accounting for 19.99%; her CD4+/CD8+ lymphocyte ratio was 2.49%.

On the 5th day after admission, the peripheral blood culture of the patient was positive for *Cellulosimicrobium cellulans*, and her vancomycin trough concentration was 3.56 mg/L. The dose frequency of vancomycin was increased (1 g, q8h), and her repeated trough concentration was 20.76 mg/L. There was no improvement in the patient’s consciousness, and lumbar puncture was done again. Her CSF pressure was 350 mmH_2_O, leukocyte count in the CSF was 27 × 10^6^/L, 5% multiple nucleated cells and 95% single nucleated cells, and her erythrocyte count was 0 × 10^6^/L. The peripheral blood culture results were positive for *Cellulosimicrobium cellulans* for 3 consecutive days. The patient eventually died of circulatory failure 13 days after admission.

### Microbiology

**Fig. 3:**
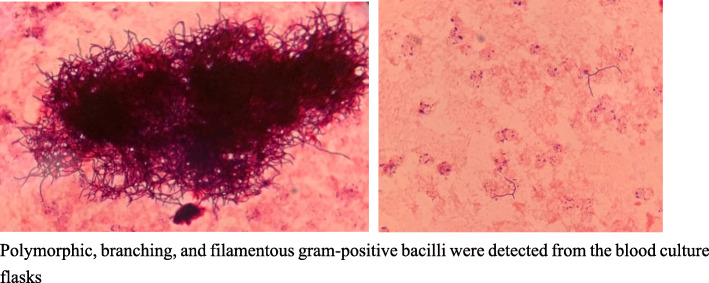
*C. cellulans* Gram staining from the positive aerobic blood culture bottle: Polymorphic, branching, and filamentous gram-positive bacilli were detected from the blood culture flasks

**Fig. 4:**
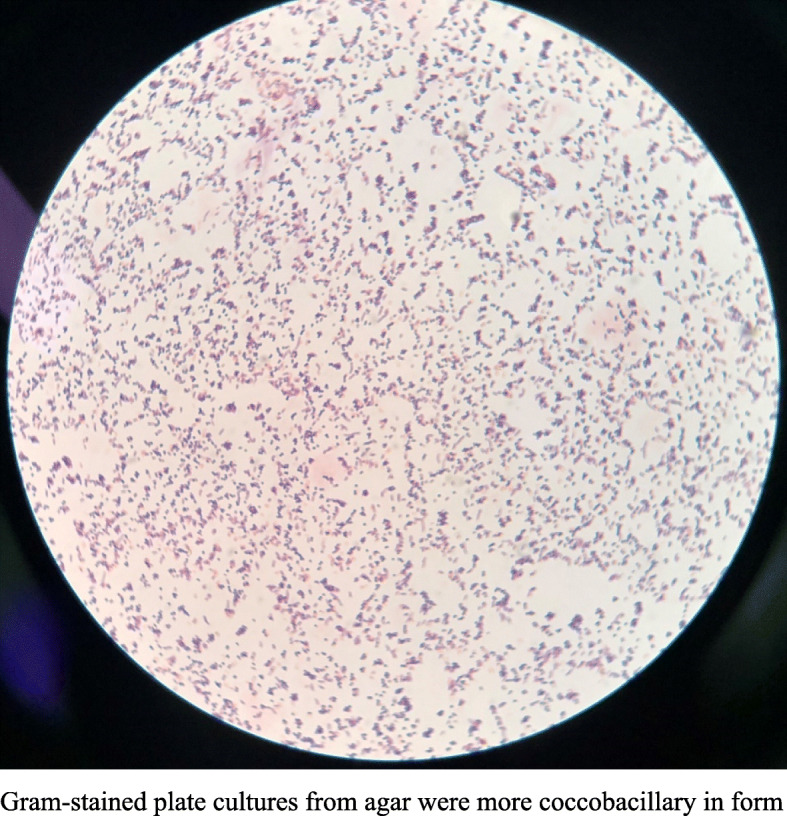
*C. cellulans* Gram staining from colonies isolated on sheep blood agar: Gram-stained plate cultures from agar were more coccobacillary in form

**Fig. 5:**
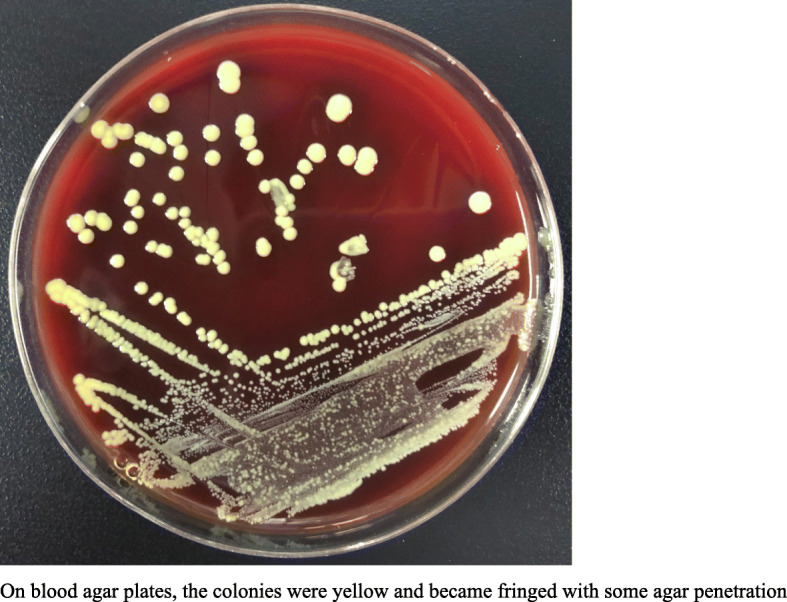
*C. cellulans* growth on a 5% sheep blood agar plate: On blood agar plates, the colonies were yellow and became fringed with some agar penetration

Aerobic and anaerobic blood cultures were positive for bacteria after 2 days of incubation. Polymorphic, branching, and filamentous gram-positive bacilli were detected in the blood culture flasks, whereas gram-stained agar plate cultures were more coccobacillary in form (Figs. 3, 4). On the blood agar plates, the colonies were yellow and became fringed with some agar penetration (Fig. 5), which is consistent with the observations of Marie-Claire Rowlinson et al. [1]. We further identified the bacteria as “*Cellulosimicrobium cellulans*” by Vitek MS mass spectrometry. Antimicrobial drug susceptibility tests were performed using our in-house paper-diffusion method to detect the inhibition ring, as reported below: levofloxacin 17 mm, ceftriaxone sodium 12 mm, vancomycin 28 mm, compounded cotrimoxazole 38 mm, rifampicin 20 mm, and tetracycline 22 mm. In the absence of clinical and laboratory standards institute (CLSI) interpretive guidelines, the organisms were considered sensitive due to their large inhibition rings.

## Discussion and conclusion

*Cellulosimicrobium cellulans* is a gram-positive filamentous bacterium that is a clinically rare pathogen, and there is much confusion about its classification. It was newly defined in 2001 as *Cellulosimicrobium cellulans* [2], of Actinobacteria class, Actinomycetales order, Micrococcineae suborder, Cellulosimicrobium genus.

*Cellulosimicrobium cellulans* is mainly found in soil and wastewater, and it has been shown that this strain can produce β-1,3 glucan, which is antagonistic and preventive toward plant fungal pathogens; thus, it can be used as a preventive strain for plant diseases [3]. It is relatively non-toxic and rarely causes human-associated infections, with only approximately 30 cases reported so far. In the reported cases, infections mainly occurred in immunocompromised hosts (human immunodeficiency virus infection, tumour-induced immunosuppression, bone marrow and solid organ transplantation, end-stage renal disease), patients with medically relevant implants (central venous catheters, transabdominal catheter, ventriculoperitoneal shunts and prostheses) and newborns. The types of infection include peritonitis, meningitis, endocarditis, endophthalmitis, catheter-associated bacteraemia, prosthetic joint infection, pyelonephritis, soft tissue infection, and suppurative arthritis [1, 4–17].

This is a case of an immunocompetent, middle-aged, rural woman, infected with *C. cellulans* without implants that caused infective endocarditis and intracranial infection. The patient started with a febrile headache that progressed to impaired consciousness after more than 20 days, and mitral valve vegetative growth was seen in transoesophageal ultrasonography. Her cranial MRI + MRA suggested the possibility of a haemorrhagic focus in the right occipital leaflet, her bilateral posterior cerebral arteries were slender, and her blood cultures were positive for *C. cellulans*. Although we did not obtain culture results from the mitral valve vegetations and intracranial blood vessels, according to the principle of disease monism, we believe that the pathogen of the mitral valve vegetations was *C. cellulans*, that the haemorrhage focus of the right occipital leaflet was caused by bacterial embolism cerebral infarction, and that the patient’s bilateral posterior cerebral arteries were also affected by bacterial destruction, which is consistent with the clinical presentation of the patient.

The cause of the consciousness disturbance in this patient also needs to be differentiated with reference to other gram-positive bacterial infections, such as tetanus infection and Nocardia infection. Tetanus is a specific infection in which *Clostridium tetani* invades the body through skin or mucosal wounds, grows and multiplies in an oxygen-deficient environment, and produces toxins that cause spasticity. Tetanus toxin mainly attacks the motor neurons in the nervous system, so the clinical features include closed teeth, paroxysmal spasm and compulsory spasm. The spasms can be triggered by mild stimuli such as light, sound, contact, drinking water, etc., or they can be spontaneous. The patient showed no muscle spasms and had no clear history of trauma, so a diagnosis of tetanus infection was ruled out.

Nocardia is a gram-positive aerobic filamentous bacterium that is widespread in soil and in livestock, and it commonly causes infections in immunocompromised hosts, typically through the respiratory tract, skin, and digestive tract. People with poor farming practices are susceptible to infection. When intracranial infection is associated with haematogenous dissemination, it may manifest as headache, weakness, convulsions, confusion, and paralysis. No Nocardia was cultured from this patient’s blood samples, and no Nocardia sequence was detected by second-generation sequencing, so Nocardia infection was not considered in this patient.

In our case, although no implants were present and no obvious skin lesions were observed, we highly suspect that the patient may have been injured by a foreign object during fieldwork. Since the patient lived alone in the countryside and was unconscious upon arrival at the hospital, her relevant medical history could not be collected. The patient’s daughter said she wore rubber shoes and long-sleeved clothes most of the time when she worked in the field. Since she fell ill in July, it cannot be ruled out that she did not take protective measures due to the hot weather. It had been more than 20 days since the onset of the disease when she reached our hospital, and the causative skin wound may have healed.

Treatment of *C. cellulans* infections varies, but in more than half of the reported cases, the infected foreign bodies must be removed. Vancomycin is the antibiotic of choice in most cases, and all cases that do not require foreign body removal have included vancomycin as part of the antibiotic regimen [11, 12, 18]. Combined administration of vancomycin and rifampicin can even clear *C. cellulans* bloodstream infections without removing the central venous catheter [1]. In our case, the patient’s treatment was delayed, she was already suffering from severe neurological impairment, and therefore, ultimately, she had a poor prognosis.

The patient had three consecutive positive blood cultures for *C. cellulans,* but there was evidence of *C. cellulans* sequences for two samples in the second-generation sequencing of her peripheral blood, and no bands were detected in her CSF. Only *Bacillus subtilis* (sequence number 3563) and *Staphylococcus aureus* (sequence number 1358) were detected in the second-generation cerebrospinal fluid sequencing. The now highly regarded second-generation sequencing seems to have limitations for specific strains. Due to the low content of intracellular bacteria released into body fluids and the low efficiency of nucleic acid extraction of fungi due to their thicker cell walls, there are often low clinical detection rates and low sensitivity. Rare pathogens and reduced bacterial populations after treatment may be interpreted by second-generation sequencing as background bacteria. When interpreting the test report, it is necessary to judge whether it is colonization bacteria, background bacteria or pathogenic bacteria based on the sample type, microbial background, clinical characteristics of the patients, traditional pathogen detection reports and auxiliary examination.

The epidemiology and pathogenicity of *C. cellulans* infections are of increasing importance in clinical microbiology. We should be aware of these opportunistic pathogens, as the number of infections they cause may increase as immunosuppressed patients survive longer and the use of medical implants increases.

## Data Availability

The data that support the findings of this study are available from Huifang Zhang, but restrictions apply to the availability of these data, which were used under license for the current study, and therefore are not publicly available. Data are, however, available from the authors upon reasonable request and with the permission of Huifang Zhang.

## Abbreviations

CRP: C-reactive protein
CSF: Cerebrospinal fluid
EEG: Electroencephalogram
GCS: Glasgow coma scale
MRA: Magnetic resonance angiography
MRI: Magnetic resonance imaging
